# Hand Osteoarthritis: investigating Pain Effects of estrogen-containing therapy (HOPE-e): a protocol for a feasibility randomised placebo-controlled trial

**DOI:** 10.1186/s40814-021-00869-1

**Published:** 2021-06-24

**Authors:** Ioana R. Marian, Megan Goff, Jennifer A. E. Williams, Malvika Gulati, Mae Chester-Jones, Anne Francis, Marion Watson, Tonia L. Vincent, Sue Woollacott, Charles Mackworth-Young, Victoria Glover, Dominic Furniss, Matthew Gardiner, Sarah E. Lamb, Katy Vincent, Vicki S. Barber, Joanna Black, Susan J. Dutton, Fiona E. Watt

**Affiliations:** 1 Oxford Clinical Trials Research Unit, Centre for Statistics in Medicine, Nuffield Department of Orthopaedics, Rheumatology, and Musculoskeletal Sciences (NDORMS), University of Oxford, Oxford, OX3 7LD UK; 2 Centre for Osteoarthritis Pathogenesis Versus Arthritis, Kennedy Institute of Rheumatology, NDORMS, University of Oxford, Oxford, UK; 3 Oxford Clinical Trials Research Unit (OCTRU), Nuffield Department of Orthopaedics, Rheumatology, and Musculoskeletal Sciences (NDORMS) University of Oxford, Oxford, OX3 7LD UK; 4 Rheumatology Department, Charing Cross Hospital, Imperial College Healthcare NHS Trust, London, UK; 5 Nuffield Orthopaedic Centre, Oxford University Hospitals NHS Foundation Trust, Oxford, UK; 6 Centre for Clinical Vaccinology and Tropical Medicine, University of Oxford, Churchill Hospital, Old Road, Oxford, OX3 7LJ UK; 7 White Horse Medical Practice, Faringdon Medical Centre, Oxfordshire, UK; 8 Wexham Park Hospital, Frimley Health NHS Foundation Trust, Frimley, UK; 9 Centre for Rehabilitation Research, NDORMS, University of Oxford, Oxford, UK; 10 College of Medicine and Health, Institute for Health Research, University of Exeter, St Luke’s Campus, Heavitree Road, Exeter, EX1 2LU UK; 11 Nuffield Department of Women’s and Reproductive Health, University of Oxford, Oxford, UK; 12 Centre for Sport, Exercise and OA Research Versus Arthritis, University of Oxford, Oxford, UK; 13 Centre for Inflammatory Disease, Department of Immunology and Inflammation, Imperial College London, Commonwealth Building, Hammersmith Campus, Du Cane Road, London, W12 0NN UK

**Keywords:** Hand osteoarthritis, Estrogen, Hormone replacement therapy, Clinical trial, Feasibility

## Abstract

**Background:**

Hand osteoarthritis (OA) is a common condition, causing pain, stiffness and reduced quality of life. Incidence is higher amongst women, particularly around the age of the menopause. Whilst the relationship between sex hormones and OA has been studied in vitro, in epidemiological studies and in clinical trials of hormone replacement therapy (HRT), this study is the first to investigate the effect of estrogen-containing therapy on hand pain in post-menopausal women with symptomatic hand OA in a randomised study design.

**Methods:**

This is a feasibility study of a double-blinded placebo-controlled intervention with 1:1 randomisation to either a combination of conjugated estrogens 0.45 mg and bazedoxifene acetate 20 mg (Duavive) or placebo. The target population is post-menopausal women with symptomatic hand OA, aiming to recruit 60–90 study participants. The primary objective is to assess the feasibility of a future fully powered randomised controlled trial (RCT). Participants will take the study medication for 24 weeks and be followed up for 28 weeks after randomisation. The primary outcomes used to determine feasibility are eligible participant identification rates and routes; recruitment, randomisation and retention rates of eligible participants; study medication compliance; and the likelihood of unintentional unblinding. Secondary outcomes include measures of hand pain, function, appearance and menopausal symptoms. An end of study questionnaire and focus groups will help to refine the final protocol for a full study.

**Discussion:**

Identifying new treatments for symptomatic hand OA is a recognised research priority. The study will help us to understand whether there are sufficient interested and eligible individuals in this target population who would consider HRT for their hand symptoms. It will provide proof-of-concept RCT data on the effects of HRT on hand pain and other clinically relevant outcomes in this population. The study will gain valuable information on the feasibility of a full RCT and how best to run this. The findings will be published in a peer-reviewed journal and presented at a relevant conference.

**Trial registration:**

ISRCTN12196200 registered on 15 January 2019.

**Supplementary Information:**

The online version contains supplementary material available at 10.1186/s40814-021-00869-1.

## Background

Hand osteoarthritis (OA) affects approximately 2 million people in the UK. The most common symptoms are hand pain, stiffness and functional difficulties. This can affect independence and the ability to work with associated negative health and social consequences [1, 2]. Typically characterised by an early, painful inflammatory phase and bony remodelling, the condition may involve the interphalangeal joints of the thumb and the fingers (proximal and distal interphalangeal joints) and the base of the thumb [3]. Patients may have only interphalangeal joint involvement, only base of thumb involvement or both.

Recommendations for the management of hand OA include general advice on joint protection, hand exercises, splinting, analgesia (such as topical anti-inflammatory gel) and sometimes intra-articular steroid injections (although their routine use is somewhat controversial) [4–6]. Whilst these treatments are often helpful, their effectiveness is frequently modest and they are not suitable for the entire population with hand OA [7–9]. Surgery remains an option in severe cases of hand OA [8]. Around 90% of those seeking specialist care for symptomatic hand OA are female [10]. Six hundred twenty thousand women aged 45–64 sought treatment for hand OA within UK primary care over a 7-year period [11]. The incidence of hand OA is higher in women than men at all ages; however the greatest relative risk compared with men is at the age of 50–55 years, which is around the age of menopause, a time of hormonal change and progressive estrogen deficiency [12–15].

There is evidence from a number of other sources that estrogens may be important in OA. Connective tissues including articular cartilage and bone have estrogen receptors and make aromatase, which synthesises estrogen in local tissues. Anti-estrogens such as aromatase inhibitors used to treat breast cancer can precipitate musculoskeletal pain or flares of symptomatic OA [16, 17]. Times of low estrogen can be associated with pain vulnerability in women, potentially exacerbating pain in the context of disease [18, 19].

Women may take hormonal replacement therapy (HRT) to control menopausal symptoms such as hot flushes, sweats and gynaecological symptoms associated with estrogen deficiency. For women with an intact uterus, HRT formulations must include progestogens or selective estrogen receptor modulators (SERMs) in addition to estrogens; this is to reduce the risk of endometrial hyperplasia and cancer [20]. SERMs are a group of agents which bind to estrogen receptors [21]. The benefits of HRT need to be considered in the context of individual risk factors, particularly female cancers and thromboembolic disease [22, 23]. First time prescription of HRT to women who are more than 10 years post-menopause or over 65 years of age is inadvisable and may have accounted for much of the morbidity in earlier studies, as reflected in the current prescribing guidelines [24–27].

To date, there have been no randomised controlled trials (RCTs) or proof-of-concept studies testing HRT in individuals with symptomatic hand OA. Observational HRT studies using varying combinations of hormones give conflicting results, perhaps because of confounding factors in those with musculoskeletal symptoms seeking or commencing HRT [13]. In post hoc analysis from large HRT clinical trials, there was evidence for a beneficial effect on musculoskeletal pain in those receiving conjugated equine estrogens (CEE), some of whom probably had OA [28]. Similarly, when studying hip and knee OA in HRT clinical trial data, modest protection from hip and also knee replacement were seen in those receiving CEE alone [29, 30]. Effects of HRT on hand OA were not specifically examined in these trials. However beneficial effects of HRT in hand OA are suggested by some epidemiological study data [31, 32].

SERMs could also have beneficial effects in OA [33]. Unlike ‘standard’ HRT, which tends to give conflicting results, consistent improvements have been reported with SERMs in animal models of OA [33–35]. In a single reported clinical trial, raloxifene improved back and knee pain, some of which was attributable to OA [36]. Newer SERMs such as bazedoxifene have a better safety profile than early-generation SERMs such as raloxifene. However, unwanted effects such as worsening post-menopausal flushing still limit acceptability of SERMs as lone agents.

Aiming to mitigate the issues of either compound used alone, Duavive was developed by Pfizer as a first-in-class combination of CEE and bazedoxifene. Duavive was approved in the US and the EU in 2014 for symptoms of estrogen deficiency. It reduced hot flushes and improved menopausal quality of life with a good safety profile [37, 38]. This combination showed no significant breast or endometrial risk compared with placebo after 2 years in several large phase III trials [39].

We aim to test the hypothesis that Duavive can improve average hand pain in post-menopausal women with symptomatic hand OA. We will not test the individual components (estrogen and bazedoxifene) in view of the safety and tolerability considerations outlined above. The combination agent (or matched placebo) would be used here in all women, irrespective of uterine status, given the mechanistic hypothesis that both components (estrogen and bazedoxifene) may have effects on OA hand pain and disease. It is also possible that this estrogen-bazedoxifene combination may be more acceptable and more effective than either treatment alone. Given a lack of precedent for RCTs in this area and uncertainties regarding recruitment, retention and proof-of concept data (including estimated effect size), a feasibility study was designed. The Hand Osteoarthritis: investigating Pain Effects of estrogen-containing therapy (HOPE-e) feasibility RCT aims to address these uncertainties and to inform the design of a future adequately powered multicentre randomised trial.

## Methods

### Study design

HOPE-e is a parallel group, double-blind randomised placebo-controlled interventional study to test the feasibility of using a licensed form of HRT (Duavive) for an alternative indication. The study examines the feasibility of a full trial, including recruitment; acceptability of randomisation; acceptability and tolerability of the medication and tests; the selection and acceptability of the proposed outcomes. Patient and public involvement (PPI) actively informed the rationale, design and development of protocol and patient facing materials of this study. Similarly, an end of study questionnaire and focus group delivered after week 28 will give study participants the opportunity to contribute to the refinement of the procedures for a future definitive trial.

### Primary objective

To assess the feasibility of a future fully powered RCT of Duavive in post-menopausal women with hand OA based on the following outcomes:

- Eligible participant identification rates and routes
- Recruitment, randomisation and retention rates
- Compliance (participant reported)
- Likelihood of unblinding

### Secondary objectives

To refine outcomes for the full study and generate proof-of concept data (including estimated effect size) on whether estrogen-containing therapy improves hand pain and other secondary outcomes in post-menopausal women with hand OA. Secondary outcomes will assess hand pain and function, menopause symptoms and joint appearance. Average hand pain will be recorded daily over the 14 days preceding each study visit, as well as the recalled average hand pain for the past 14 days at the study visit. Daily pain rating will either be by smart phone or by paper diary, depending on participant preference (see “Data collection methods” section). All outcome measures to address the primary and secondary objectives are listed in Table 1.

**Table 1:** Outcome measures

Primary objectives outcome measures	Secondary objectives outcome measures		
	Pain and function outcomes	Menopause symptoms	Joint appearance
• Rates of eligible participant identification (screening period) • Rates of recruitment/randomisation from different sources (screening period) • Retention rates (throughout study) • Study medication compliance ^a^ • Bang’s Blinding Index (likelihood of unblinding) [40] ^a^	• Average hand pain (rated over 14 days preceding the study visit)^c a^ • Pain manikin–capturing pain in other joints in the 4 weeks preceding the study visit^a^ • Functional Index for Hand OsteoArthritis (FIHOA) questionnaire [41, 42]^b^ • Grip strength, by Jamar dynamometer [43]^b^ • EQ-5D-5L questionnaire [44, 45]^b^	• Menopause-specific Quality of Life (MENQOL) questionnaire (Intervention 1-month recall version) [38, 46, 47]^b^ • Greene Climacteric Scale [48]^b^	• Cosmesis score (single question, Michigan Hand Questionnaire) [49]^b^ • Investigator-recorded tender and swollen joint counts ^a^ • Photographic recording of swollen hand joints ^a^

^a^See Fig. 1 for collection time points

^b^See “Questionnaires” in Table 2 for collection time points

^c^Proposed primary outcome for full trial

The protocol follows the Standard Protocol Items: Recommendations for Interventional Trials (SPIRIT) guidelines [50]. The SPIRIT checklist is provided as Supplementary Table 1 in the Additional files.

### Study setting

This feasibility study will be undertaken at a small number of sites, including a range of primary and secondary care National Health Service (NHS) sites in the UK. Participants will also be recruited directly from the community, through participant identification centres and via a number of advertising methods. A list of the study sites can be provided on request from the HOPE-e study office.

### Eligibility criteria

In order to be eligible for the study, patients must comply with all of the following:

- Able to give written informed consent
- Female aged 40–65 years old
- In those with an intact uterus: at least 12 months of spontaneous amenorrhea (without any menstrual bleeding in last 12 months) and last menstrual period not more than 10 years ago
- In those who have undergone hysterectomy or are/were using an intrauterine contraceptive device with progesterone local therapy (such as Mirena^TM^): follicle stimulating hormone (FSH) ≥ 30mIU/ml) on screening blood test and a history of menopausal symptoms in the last 1 to 10 years, in keeping with appropriate timing of menopausal status
- Hand pain, aching or stiffness on most days in the last 3 months and fulfils American College of Rheumatology clinical diagnostic criteria for hand OA (3 or more of the following) [51]:
  - ○ Hard tissue enlargement of two or more of the following joints: 2nd or 3rd distal interphalangeal joints (DIPJ), 2nd or 3rd proximal interphalangeal joints, first carpometacarpal joints
  - ○ Hard tissue enlargement of two or more of the DIPJs
  - ○ Fewer than three swollen metacarpophalangeal joints
  - ○ Deformity of at least one of the joints listed in the first point
- Minimum of two painful hand joints of any type (interphalangeal joints (IPJ) or base of thumbs)
- Hand pain that has not responded adequately to National Institute for Health and Clinical Excellence (NICE) core guidance for the management of OA [27], including the use of paracetamol or non-steroidal anti-inflammatory topical gel, except where there is contraindication or intolerance
- Average hand pain is reported as typically more than 4 out of 10 in severity, or average hand pain in the last 7 days of 4/10 or more on a visual analogue scale
- Able and willing to comply with all study requirements

Participants may not enter the study if any of the following apply:

- Other cause of hand pain, including inflammatory arthritis, connective tissue disorder, chronic pain, or alternative clinical diagnosis such as tenosynovitis or carpal tunnel syndrome
- Pregnancy or breast feeding, or risk of this during study
- Use of one or more prohibited treatments within specified timeframe, or not willing to avoid treatment for the duration of the study (see additional details in Supplementary Table 2)
- Presence of one or more medical contraindications to the use of systemic hormonal replacement therapy (see additional details in Supplementary Table 2)
- Any other significant or uncontrolled disease or disorder which may either put the participants at risk because of taking part in the study, or may influence the result of the study, or the participants’ ability to participate
- Participants involved in another research trial involving an investigational product in the past 8 weeks

### Blood and urine testing

Blood samples will be taken at screening as part of the eligibility process and at weeks 4, 12, and 24 for monitoring the safety of therapy. A mid-stream urine sample will be tested by dipstick for blood, protein and glucose at screening. The urine sample will be used to perform beta human chorionic gonadotropin (β-HCG) testing by test stick to exclude pregnancy, at the investigator’s discretion. For safety reasons, at all other visits, a urine dipstick for blood, protein, and glucose or urinary β-HCG testing can be performed at the discretion of the investigator.

### Pregnancy and contraception

Duavive is only licensed for use in postmenopausal women and is contraindicated in women who are, or may become, pregnant. If pregnancy occurs during treatment, Duavive should be withdrawn immediately. Individuals entering the study must be at least 1 year after natural cessation of periods. A urinary pregnancy test will be conducted at the screening visit and subsequent visits at the investigator’s discretion, for example in those who are within 2 years of final menstrual period, and/or under the age of 45. Where there is clinical concern, those individuals will be advised to use two reliable methods of barrier contraception.

### Interventions

In the intervention group, conjugated estrogens 0.45 mg-bazedoxifene acetate 20mg (Duavive, Pfizer) will be administered orally as a tablet once daily for 24 weeks. There is no possibility for dose modification during the study. A matched placebo was manufactured and packaged by MODEPHARMA Ltd., UK. The placebo and the study medication tablets are in blister packs and will be placed in opaque plain box packaging at the time of dispensing. Members of the research team and staff conducting participant visits will not handle the study medication, and participants will be asked to return unused medication to the local pharmacy for safe disposal.

Study participants, care providers, study team (except the statistician and one other member of the study team) and outcome assessors will be blinded to intervention.

### Participant timeline

**Fig. 1:**
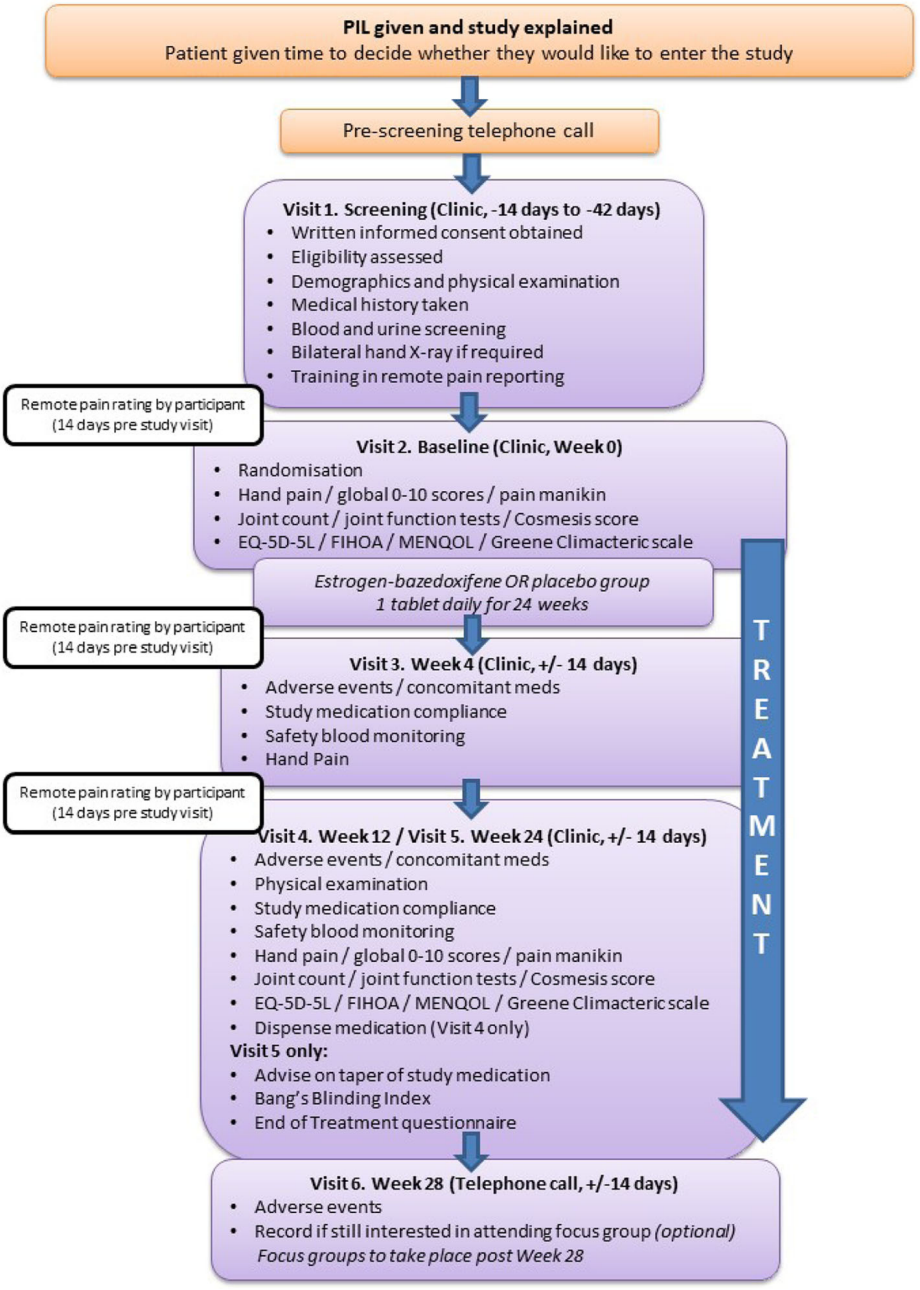
Study flow chart

The timeline for participants is illustrated in Fig. 1.

### Recruitment

Potentially eligible participants will be identified and approached via multiple routes in order to maximise recruitment. These include discussion at routine clinic appointments (primary care and different specialties in secondary care including rheumatology, orthopaedics, hand surgery and therapies); contacting individuals who have registered an interest in OA research; publicity leading to self-referral, both online (websites, Twitter and other social media, Short Message Service (SMS)) and offline (posters and flyers displayed in secondary care research sites, general practice surgeries and community spaces such as physiotherapy clinics and pharmacies, community magazine advertising). Following provision of a Participant Information Leaflet (PIL) to those expressing interest, key eligibility criteria will be assessed in those verbally consenting to an initial telephone pre-screen, following which individuals will be invited to attend a screening visit in person at the nearest recruitment site.

### Consent

A medically qualified and GCP trained clinician will obtain informed consent from the patient at the screening visit at least 24 h after receiving the PIL, following patient verification of understanding of all aspects of the study and having answered all questions and documenting this by completion of the study informed consent form.

### Screening assessment

Following consent, full eligibility, including that assessed by blood test results, will be reviewed as part of the screening assessment and prior to randomisation (see Fig. 1). An X-ray of both hands is also offered at the end of screening visit to all those who appear eligible and who have not undergone a hand X-ray in the last 3 years. Although the presence of radiographic hand OA is not required for inclusion in the study, its presence or absence will be reported for participants entering the study. Should an individual fail to meet eligibility criteria at screening assessment based on a reversible criterion (e.g. initiation of oral analgesia within 4 weeks of screening visit), re-screening may be carried out if the participant agrees. If a participant fails to meet the inclusion and exclusion criteria on review of results from the screening visit, they will be withdrawn and not proceed with the study. They will be contacted with an explanation and advice as appropriate.

### Randomisation

Consenting, eligible participants will be randomised 1:1 to either Duavive or placebo at the baseline visit. Randomisation to the interventions will be carried out via a secure (encrypted) web-based randomisation service, the Oxford Clinical Trials Research Unit (OCTRU) Registration, Randomisation and Management of Product (RRAMP). Randomisation will be performed using a minimisation algorithm including a random element to ensure balanced allocation of participants across the two treatment groups, stratified by centre and the profile of painful joints (IPJ hand OA only; IPJ plus base of thumb OA; or base of thumb OA only) [52]. To prevent predictability, the minimisation algorithm will be seeded, randomising the first few participants using simple randomisation. Specific details will be stored within a randomisation and blinding plan, stored securely in the confidential statistical trial master file.

### Data collection methods

Study outcome data will be collected during five face-to-face study visits and one remote visit. Details of visit timings and outcomes are presented in Fig. 1 and Table 2; these are the following:

- Screening (eligibility)
- Baseline (randomisation, first prescription of study medication)
- 4 weeks (safety, adherence)
- 12 weeks (safety, adherence, second prescription of study medication)
- 24 weeks (primary outcome, advice on weaning study medication over subsequent 4 weeks)
- 28 weeks phone call (safety, review of weaning of study medication)

**Table 2:** Timing of visits and data collection (SPIRIT)

	Visits/time points					
	1	2	3	4	5	6
Procedures	Screening	Baseline				Follow-up
	Maximum 42 days	Week 0	Week 4	Week 12	Week 24	Week 28
Informed consent	x					
Inclusion/exclusion	x	x	x	x	x	
Demographics	x					
Medical history	x					
Concomitant medication	x	x	x	x	x	
Hand pain 0–10 NRS	x	x	x	x	x	
Pain manikin		x		x	x	
Physical examination	x		x	x	x	
Vital signs	x		x	x	x	
Blood screening	x					
Urine dipstick	x	X^a^	X^a^	X^a^	X^a^	
Urine pregnancy test	X^a^	X^a^	X^a^	X^a^	X^a^	
Bilateral hand X-ray *(if applicable)*	x					
Hand pain rating (remote)	Training	x	x	x	x	
Randomisation		x				
Prescription/dispensing		x		x		
Reported study medication compliance			x	x	x	
Advice on barrier contraception		X^a^	X^a^	X^a^	X^a^	
Joint count		x		x	x	
Questionnaires		x		x	x	
Photographs of swollen hand joints		x		x	x	
Grip strength		x		x	x	
Safety blood monitoring ^c^			x	x	x	
Safety/adverse events			x	x	x	x
Advice on weaning off study medication					x	
Bang’s Blinding Index					x	
End of treatment questionnaire					x	
Focus group^b^ *(optional)*						

^a^At discretion of investigator

^b^After last participant has completed visit 6

^c^Includes creatinine, urea and electrolytes, liver function, full blood count and C-reactive protein

We anticipate that any effects of the intervention on symptoms would be evident by between 4 and 24 weeks after initiation; therefore, the main outcomes will be collected at baseline, week 12 and week 24. Face-to-face visits will be necessary to assess for Adverse Events (AEs), provide new prescriptions and perform safety blood tests in addition to collection of other data (Fig. 1 and Table 2).

There will be a 28-week phone call to review progress following weaning of study medication after 24 weeks (best practice is to avoid abrupt cessation of HRT and this allows for this) and collect/assess any AEs. Some consenting participants will be randomly selected to attend one of two optional focus groups after week 28.

Outcome data will be collected via Paper Case Report Forms completed by the investigator and participant. Remote daily rating of hand pain in the 14 days prior to a study visit will be captured on paper diaries or via the use of a single response online questionnaire (Limesurvey) sent to the participant’s smart phone via SMS, depending on the participant’s preference.

### Data management

All study data will be entered into a secure password-protected database (OpenClinica®), except for the electronic remote daily pain rating which is automatically stored in the study database. All hand photographs, audio recordings and transcripts of focus groups will be kept in a linked anonymised format and stored securely. Audio recordings will be deleted at the end of the study. A Data Management Plan which includes references to confidentiality, access and security arrangements has been produced for the study and is available on request from the HOPE-e study office.

### Concomitant medications

Concomitant medication will be recorded at each visit. The participants will be immediately discontinued from the study medication if they commence any other systemic hormonal therapy (such as another form of systemic HRT or the oral contraceptive pill), any form of anti-estrogen medication or initiation of a liver-enzyme inducing agent. The medications or interventions described in Table 3 will be discouraged during the study; their use will be documented, but the participant will be allowed to remain in the study.

**Table 3:** Concomitant medications

• Intra-articular steroid, particularly when into the hands or within 3 months of the week 24 visit • Oral steroid, particularly when for longer than 5 days consecutively or within 6 weeks of the week 24 visit • Intramuscular steroid at any point during the study • Use of intra-articular hyaluronan to a hand joint at any point during the study • New prescription of non-steroidal anti-inflammatory drugs or other analgesic, particularly when within 4 weeks of the week 24 visit • Initiation of treatment such as glucosamine or chondroitin at any point • Initiation of hand exercises or other relevant non-pharmacological therapy at any point	

### Compliance with the study medication

Clinically significant non-compliance will be defined as more than 14 days of missed medication in any calendar month, following which the participant will be withdrawn from study medication but invited to continue study follow-up.

### Breakthrough pain relief medication

Participants may experience flares of hand pain during the study. During this time, they should take their usual analgesic medications as required. If possible, no new medications should be commenced for pain in the hand or elsewhere. Where paracetamol is not usually taken already, participants will be instructed that they can use paracetamol up to 1 g four times daily for the relief of breakthrough pain if they wish, preferably avoiding taking any in the 24 h prior to a study visit. Any use of such breakthrough pain relief will be documented at each visit, and the participant asked to record this in their study paper diary.

### Statistical analysis

As this is a feasibility study not aiming to assess treatment effects but to test rates and feasibility of randomisation, a formal power calculation was not conducted. Instead we estimated 60–90 participants as the number required for the feasibility study in order to be able to accurately calculate a sample size that could detect a moderate standardised effect size of 0.3 to 0.5 in the future definitive trial [53]. Outcome data from this number of participants will be used to estimate standard deviations and confidence intervals (CIs) of the treatment estimates which will be used to inform a sample size calculation for a definitive trial.

**Fig. 2:**
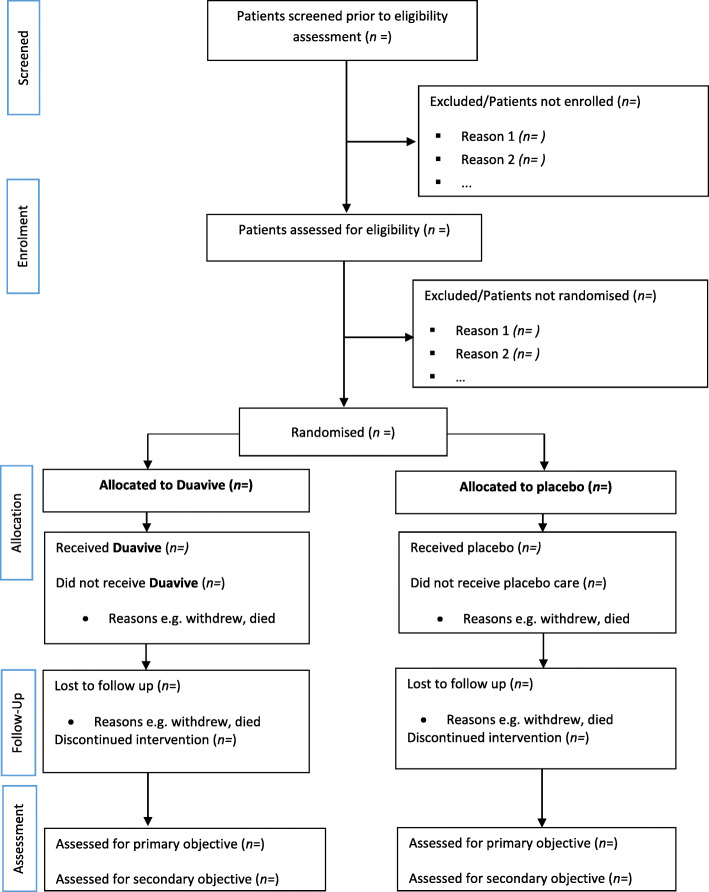
CONSORT pilot and feasibility trials flow diagram schematic

The primary analysis will evaluate the feasibility of this study design based on the outcome measures for the primary research objectives described in Table 1: rates of eligible participants; rates of recruitment/randomisation; retention rates; and study medication compliance. These outcomes will be reported on an intention-to-treat (ITT) basis for each treatment group, with sensitivity analyses conducted on a per protocol (PP) basis. The PP population will exclude any participants with protocol deviations pre-specified in the analysis plan, such as reported non-compliance with the medication or reported concomitant medications use (as per Table 3). We will identify those who completed the study PP prior to unblinding of any analyses. Mean and standard deviation, or median and interquartile range, will be presented for continuous outcomes, depending on distribution, and numbers with percentages for binary and categorical data, for all planned primary and also for secondary outcome measures. Demographic and clinical characteristics for the two groups at baseline will be presented descriptively. Results will be reported descriptively as per Fig. 2 and in accordance with the Consolidated Standards of Reporting Trials (CONSORT) extension to randomised pilot and feasibility trials [54].

The secondary objective outcome measure, average hand pain is the planned primary outcome for the future definitive trial. This outcome is scored as 0–10 on a Numerical Rating Scale and collected from participant daily pain ratings for the 14 days prior to the baseline (week 0) and the week 24 study visit. The participant preference for hand pain data collection (online questionnaire or paper diary) will be compared and reported. Average hand pain will be reported as means and 95% CI based on a multivariate linear regression model with adjustment for the minimisation factors: study site (3 levels), and pattern of involvement of painful joints (IPJ hand OA only, IPJ plus base of thumb OA, or base of thumb OA only). This will be compared descriptively with the recalled average hand pain over the last 14 days at each visit, in terms of completion rates and differences between these measures. The change in average hand pain from baseline to 24 weeks and the difference in the means together and the corresponding 95% CI will also be reported for each treatment group and overall. No formal statistical testing will be undertaken, as the study is not powered for this purpose. Additional analyses will include blood monitoring and safety data; pre-screen and screen-out rates; willingness to be randomised if eligible; and other measures of acceptability, including retention rates both overall and by recruitment site; response in those with IPJ hand OA only, IPJ and base of thumb OA, and base of thumb OA only; and those with or without evidence of radiographic hand OA. Further analysis outside of this primary analysis plan may be necessary to fully understand the feasibility of this study and to inform the future definitive trial. If this occurs, such analysis will be described in full in any results publication arising from this feasibility study. Reasons for participant unblinding; missing data; withdrawals or loss to follow-up will be carefully considered and reported by treatment group and patterns of ‘missingness’ will be explored.

Data will be analysed using an appropriate validated statistical software such as Stata, StataCorp LP, USA [55]. No comparative interim analyses will be performed. A trial statistician will contribute to the statistical aspects of the study.

### Data monitoring

A monitoring plan has been developed and will be followed throughout the study. A Trial Steering Committee (TSC) will oversee the overall conduct of the study and make recommendations on the feasibility of a full trial following completion. The TSC will comprise of invited expert members, including at least one medically qualified person, and patient representatives. A separate medically qualified Safety Oversight Clinician (SOC) (with appropriate clinical experience and independent to the study) will be appointed to safeguard the interest of the study participants. The SOC can advise the chair of the TSC at any time, if in their view, the study should be stopped due to concerns over participant safety.

Stop-go criteria for progression to a future definitive trial were predefined at the outset of the study. These were (i) recruitment of sufficient participants in a defined period (pre-specified as ≥ 30 participants across all sites in 18 months, or, proportionate to this e.g. ≥ 22 participants in 12 months); (ii) a drop-out rate of ≤ 30% of randomised individuals; and (iii) acceptability to the majority of participants, including acceptable rates of AEs. The TSC will make recommendations on the feasibility of a future definitive trial based in part, on all three of these pre-defined criteria being met.

### Withdrawals

Participants have the right to withdraw from the study at any time and without giving reason. Participants asking to stop treatment or unable to tolerate the treatment will be withdrawn from the treatment. In the event of ineligibility (either arising during the study or retrospectively having been overlooked at screening) the participant will be withdrawn. The investigator may discontinue a participant from the study medication at any time if the investigator considers it necessary for any reason (e.g. unblinding; pregnancy; surgical procedures to the hand; AEs necessitating withdrawal of HRT; new diagnosis of cancer). Withdrawn participants will be given advice on weaning treatment if relevant, depending on the safety considerations. Those withdrawn from the study will be invited to an optional end of study visit at the time of their withdrawal.

### Safety

All AEs will be collected at all visits as part of assessing the acceptability of the medication. These data will be summarised and reviewed by the SOC and the TSC. However, as this is a feasibility study with a licensed medication and an established safety profile, only rare events (≥ 1/10,000 to < 1/1000, i.e. venous thromboembolic events including pulmonary embolism, retinal vein thrombosis, deep vein thrombosis and thrombophlebitis) and any other serious event deemed to be related and unexpected will be reported to the sponsor.

Worsening of the underlying disease as a direct result of the study medication will be reported as a Serious Adverse Reaction (SAR).

All serious adverse events (SAEs) will be reported to the trials unit within 24 h of the study team becoming aware. The relationship of each AE to the study medication will be determined by a medically qualified individual. These SAEs will also be reported to the Research Ethics Committee that gave a favourable opinion of the study if the chief investigator believes the event was ‘related’ (i.e. resulted from administration of any of the research procedures) and ‘unexpected’ in relation to those procedures.

In the event of a SAR or any suspicion of a SAR, the study medication will be stopped immediately. Stopping medication will also be considered for any AEs as part of the review process.

The following instances will also be reported as an AE and will need further investigation if they occur:

1. Those who experience postmenopausal bleeding and unblinding are carried out and they are shown to be on placebo.
2. Those who experience persistent or severe bleeding, ongoing bleeding beyond 12 weeks, or new bleeding after 12 weeks of taking Duavive (may represent a SAE), depending on the underlying cause and the medical response to this.

When a participant has reported vaginal spotting or bleeding, they will be provided with a ‘breakthrough bleeding diary’ to record information on the nature and frequency of bleeding. Unblinding or withdrawal of study medication may be considered, and the advice of the study gynaecologist will be sought, particularly if further investigation is required. These events may represent a SAE, depending on the underlying cause. A trial specific instruction document has been designed to outline the review process in detail and is provided as Supplementary Figure 1 in Additional files.

### Unblinding

Emergency and non-emergency unblinding will be available via the RRAMP system 24 h a day.

Accidental unblinding (for example due to breakthrough bleeding) of the participant or the investigator may occur and will be documented in an unblinding log, and its likelihood will be assessed at the end of the study. Accidental unblinding will not affect the majority of primary feasibility outcomes and participants would therefore be encouraged to continue their medication and complete the study. If a participant’s allocation is unblinded for safety reasons during the study, the participant will be withdrawn from medication at that point but invited to attend follow-up study visits and complete the study.

### Auditing

Direct access will be granted to authorised representatives from the sponsor, host institution and the regulatory authorities to permit study-related monitoring, audits and inspections.

### Ancillary and post-trial care

There is no provision for continuation of the medication beyond the end of the study. If participants are eligible for HRT due to symptoms which are within standard indications for HRT use, the participant will be advised to discuss this with their GP.

### Dissemination policy

Reporting of the HOPE-e study will be in line with the CONSORT extension for randomised pilot and feasibility trials guidelines [54] and will be agreed at TSC meetings. The results of the study will be published and disseminated via oral reports at international meetings and on University of Oxford websites. Study results will be disseminated to study participants via letters following end of study and analysis.

### Protocol amendments

Favourable opinion of two substantial amendments was obtained on 9 August 2019 and 18 December 2019. A history of the protocol amendments is presented in Supplementary Table 3.

### Confidentiality

The study staff will ensure that the participants’ confidentiality is maintained. The study will comply with the General Data Protection Regulation and Data Protection Act, which requires data to be anonymised as soon as it is practical to do so. Any communication containing participant identifiable data from sites will be by NHS.net email or secure encrypted fax. The participants will be identified only by a participant code on all study documents and study database. All documents will be stored securely and only accessible to authorised personnel.

### Declaration of interests

Duavive was purchased through the Oxford University Hospitals NHS Foundation Trust Pharmacy Purchasing and Distribution Unit and distributed to sites. The selection of study medication was on academic grounds. Pfizer had no part in the conception, funding or provision of study medication for this study.

## Discussion

There is an ongoing search to identify pharmacological therapies which improve pain or target underlying disease processes in OA, slowing its progression. The preponderance of symptomatic OA in post-menopausal women, with a particularly close association between the age of menopause (~ 50 years) and the incidence of hand OA, makes targeting estrogen-related pathways an attractive proposition in this disease.

Not all women can take HRT safely. Despite a large amount of supporting safety data since the Women’s Health Initiative in appropriate age groups nearer to menopause, there remains distrust amongst some of the public, the media and parts of the medical profession about the use of HRT. Considering attitudes to medications in the acceptability and feasibility of this intervention is therefore critical, particularly in terms of the perceived risk and benefit of taking a medication in this target population.

The choice of agent in the study was important. Here, we have chosen a combined oral HRT: an estrogen and a SERM. By arguing mechanistically for both components, we have justified administration to all participants, irrespective of uterine status (usually those with hysterectomy would just take unopposed estrogen as HRT). We have selected an agent based on the preclinical/clinical data supporting likelihood of effect (of both components), the reported lower incidence of breakthrough bleeding with this agent and because participants in our discussion group were attracted by this newer agent, especially as both components may have a positive effect. If an efficacy signal is seen (although not powered for this), this study would not demonstrate which element of the agent was mediating this. Subsequent mechanistic studies would be needed to deconvolute any effect.

Much of our evidence implicating the menopause in the onset of hand OA and for estrogen as a relevant target to date is circumstantial: from epidemiological studies, observational studies and convenience data from large HRT trials. There is further insight from a recently published report (since the development of our protocol) from electronic healthcare data in UK Community Practice Research Datalink [15]. This analysis emphasises the close temporal link to menopause (the highest rates of hand OA were seen in the year after menopause), but not all women have a temporal association to menopause (approximately 60% appear to). The study suggests that those starting HRT near to their menopause gained the most benefit in terms of protection from hand OA. In this protocol, to adhere to good practice in HRT prescribing, we mandate that participants will be at least 1 year after final menstrual period, and that they have established hand OA clinically. Whether this will prove too late, either in terms of the course of their menopause and/or their disease course to effect change on symptoms is yet to be determined. That cessation of HRT appears to be associated with onset of OA in this study is further evidence for a modulatory role for these agents, which needs to be better understood. It is important to note that here, the agent will be weaned gradually over 4 weeks, rather than being stopped abruptly to reduce the chances of any symptom flare.

To our knowledge, the study is unique, aiming to build our knowledge of whether it is possible to intervene in a poorly understood area, that of menopause and related female musculoskeletal health issues. It seeks to assess a potential tailored intervention for a group with high unmet clinical need. It will also give us valuable information on the best outcomes in this particular group of women with the condition. Particular strengths of the study are its testing of feasibility and acceptability of different ways of collecting average hand pain, which is an established outcome measure, although one that was highlighted by participants in our PPI as inadequately measured. HOPE-e is designed to determine whether a large RCT treating symptomatic hand OA in post-menopausal women with estrogen therapy is feasible. The study opened to recruitment in May 2019 (IRAS ID 236463) and is anticipated to close in February 2021.

## Supplementary Information

**Additional file 1: Supplementary Table 1**. SPIRIT 2013 Checklist: Recommended items to address in a clinical trial protocol and related documents*. **Supplementary Table 2**. Eligibility Criteria (exclusion, further details). **Supplementary Figure 1**. Flow Chart for Postmenopausal Bleeding. **Supplementary Table 3**. Protocol Amendment History*.

## Data Availability

Direct access to research data will be granted to authorised representatives of the sponsor, regulatory authorities or the host institution for monitoring and/or auditing of the study to ensure compliance with regulations.

## Abbreviations

AE: Adverse events
β-HCG: Beta human chorionic gonadotropin
CEE: Conjugated equine estrogens
CI: Confidence interval
CONSORT: Consolidated Standards or Reporting Trials
DIPJ: Distal interphalangeal joints
FIHOA: Functional Index for Hand OsteoArthritis
FSH: Follicle stimulating hormone
GCP: Good Clinical Practice
HOPE-e: Hand Osteoarthritis: investigating Pain Effects in a randomised placebo-controlled feasibility study of estrogen-containing therapy
HRT: Hormone replacement therapy
IPJ: Interphalangeal joint
ITT: Intention-to-treat
MENQOL: Menopause-specific quality of life
NHS: National Health Service
NIHR: National Institute for Health Research
OA: Osteoarthritis
OCTRU: Oxford Clinical Trials Research Unit
PIL: Patient Information Leaflet
PP: Per protocol
PPI: Patient and public involvement
RRAMP: Registration, Randomisation and Management of Product
RCT: Randomised controlled trial
SAE: Serious adverse event
SAR: Serious adverse reaction
SERM: Selective estrogen receptor modulator
SMS: Short message service
SOC: Safety Oversight Clinician
SPIRIT: Standard Protocol Items: Recommendations for Interventional Trials
TSC: Trial Steering Committee
UK: United Kingdom
VTE: Venous thromboembolism
