# Supplementary material for: Hand Osteoarthritis: investigating Pain Effects of estrogen-containing therapy (HOPE-e): a protocol for a feasibility randomised placebo-controlled trial
Source: Pilot Feasibility Stud. 2021 Jun 24;7:133. doi: 10.1186/s40814-021-00869-1 (PMC8223359; doi:10.1186/s40814-021-00869-1)
Supplement: Supplementary file 1 — Additional file 1: Supplementary Table 1. SPIRIT 2013 Checklist: Recommended items to address in a clinical trial protocol and related documents*. Supplementary Table 2. Eligibility Criteria (exclusion, further details). Supplementary Figure 1. Flow Chart for Postmenopausal Bleeding. Supplementary Table 3. Protocol Amendment History*. [file 40814_2021_869_MOESM1_ESM.docx]

**Supplementary Table 1**. SPIRIT 2013 Checklist: Recommended items to address in a clinical trial protocol and related documents*

| Section/item | ItemNo | Description | Page |
| --- | --- | --- | --- |
| **Administrative information** | | |  |
| Title | 1 | Descriptive title identifying the study design, population, interventions, and, if applicable, trial acronym | 1 |
| Trial registration | 2a | Trial identifier and registry name. If not yet registered, name of intended registry | 3 |
|  | 2b | All items from the World Health Organization Trial Registration Data Set | Throughout |
| Protocol version | 3 | Date and version identifier | 20 |
| Funding | 4 | Sources and types of financial, material, and other support | 21, 25 |
| Roles and responsibilities | 5a | Names, affiliations, and roles of protocol contributors | 1, 25 |
|  | 5b | Name and contact information for the trial sponsor | 25 |
|  | 5c | Role of study sponsor and funders, if any, in study design; collection, management, analysis, and interpretation of data; writing of the report; and the decision to submit the report for publication, including whether they will have ultimate authority over any of these activities | 25 |
|  | 5d | Composition, roles, and responsibilities of the coordinating centre, steering committee, endpoint adjudication committee, data management team, and other individuals or groups overseeing the trial, if applicable (see Item 21a for data monitoring committee) | 18, 26 |
| Introduction |  |  |  |
| Background and rationale | 6a | Description of research question and justification for undertaking the trial, including summary of relevant studies (published and unpublished) examining benefits and harms for each intervention | 3, 4, 5 |
|  | 6b | Explanation for choice of comparators | 5 |
| Objectives | 7 | Specific objectives or hypotheses | 6 |
| Trial design | 8 | Description of trial design including type of trial (eg, parallel group, crossover, factorial, single group), allocation ratio, and framework (eg, superiority, equivalence, noninferiority, exploratory) | 6 |
| Methods: Participants, interventions, and outcomes | | |  |
| Study setting | 9 | Description of study settings (eg, community clinic, academic hospital) and list of countries where data will be collected. Reference to where list of study sites can be obtained | 7 |
| Eligibility criteria | 10 | Inclusion and exclusion criteria for participants. If applicable, eligibility criteria for study centres and individuals who will perform the interventions (eg, surgeons, psychotherapists) | 8 |
| Interventions | 11a | Interventions for each group with sufficient detail to allow replication, including how and when they will be administered | 10, 11 |
|  | 11b | Criteria for discontinuing or modifying allocated interventions for a given trial participant (eg, drug dose change in response to harms, participant request, or improving/worsening disease) | 14, 17, 18 |
|  | 11c | Strategies to improve adherence to intervention protocols, and any procedures for monitoring adherence (eg, drug tablet return, laboratory tests) | 11, 13 |
|  | 11d | Relevant concomitant care and interventions that are permitted or prohibited during the trial | 14 |
| Outcomes | 12 | Primary, secondary, and other outcomes, including the specific measurement variable (eg, systolic blood pressure), analysis metric (eg, change from baseline, final value, time to event), method of aggregation (eg, median, proportion), and time point for each outcome. Explanation of the clinical relevance of chosen efficacy and harm outcomes is strongly recommended | 1, 7, 8 |
| Participant timeline | 13 | Time schedule of enrolment, interventions (including any run-ins and washouts), assessments, and visits for participants. A schematic diagram is highly recommended (see Figure) | 13 |
| Sample size | 14 | Estimated number of participants needed to achieve study objectives and how it was determined, including clinical and statistical assumptions supporting any sample size calculations | 15 |
| Recruitment | 15 | Strategies for achieving adequate participant enrolment to reach target sample size | 11 |
| **Methods: Assignment of interventions (for controlled trials)** | | |  |
| Allocation: |  |  |  |
| Sequence generation | 16a | Method of generating the allocation sequence (eg, computer-generated random numbers), and list of any factors for stratification. To reduce predictability of a random sequence, details of any planned restriction (eg, blocking) should be provided in a separate document that is unavailable to those who enrol participants or assign interventions | 12 |
| Allocation concealment mechanism | 16b | Mechanism of implementing the allocation sequence (eg, central telephone; sequentially numbered, opaque, sealed envelopes), describing any steps to conceal the sequence until interventions are assigned | 12 |
| Implementation | 16c | Who will generate the allocation sequence, who will enrol participants, and who will assign participants to interventions | 12 |
| Blinding (masking) | 17a | Who will be blinded after assignment to interventions (eg, trial participants, care providers, outcome assessors, data analysts), and how | 11 |
|  | 17b | If blinded, circumstances under which unblinding is permissible, and procedure for revealing a participant’s allocated intervention during the trial | 19 |
| **Methods: Data collection, management, and analysis** | | |  |
| Data collection methods | 18a | Plans for assessment and collection of outcome, baseline, and other trial data, including any related processes to promote data quality (eg, duplicate measurements, training of assessors) and a description of study instruments (eg, questionnaires, laboratory tests) along with their reliability and validity, if known. Reference to where data collection forms can be found, if not in the protocol | 13 |
|  | 18b | Plans to promote participant retention and complete follow-up, including list of any outcome data to be collected for participants who discontinue or deviate from intervention protocols | 17 |
| Data management | 19 | Plans for data entry, coding, security, and storage, including any related processes to promote data quality (eg, double data entry; range checks for data values). Reference to where details of data management procedures can be found, if not in the protocol | 14 |
| Statistical methods | 20a | Statistical methods for analysing primary and secondary outcomes. Reference to where other details of the statistical analysis plan can be found, if not in the protocol | 15, 16 |
|  | 20b | Methods for any additional analyses (eg, subgroup and adjusted analyses) | N/A |
|  | 20c | Definition of analysis population relating to protocol non-adherence (eg, as randomised analysis), and any statistical methods to handle missing data (eg, multiple imputation) | N/A |
| **Methods: Monitoring** | | |  |
| Data monitoring | 21a | Composition of data monitoring committee (DMC); summary of its role and reporting structure; statement of whether it is independent from the sponsor and competing interests; and reference to where further details about its charter can be found, if not in the protocol. Alternatively, an explanation of why a DMC is not needed | 17 |
|  | 21b | Description of any interim analyses and stopping guidelines, including who will have access to these interim results and make the final decision to terminate the trial | 17 |
| Harms | 22 | Plans for collecting, assessing, reporting, and managing solicited and spontaneously reported adverse events and other unintended effects of trial interventions or trial conduct | 18 |
| Auditing | 23 | Frequency and procedures for auditing trial conduct, if any, and whether the process will be independent from investigators and the sponsor | 19 |
| Ethics and dissemination | | |  |
| Research ethics approval | 24 | Plans for seeking research ethics committee/institutional review board (REC/IRB) approval | 24 |
| Protocol amendments | 25 | Plans for communicating important protocol modifications (eg, changes to eligibility criteria, outcomes, analyses) to relevant parties (eg, investigators, REC/IRBs, trial participants, trial registries, journals, regulators) | 20 |
| Consent or assent | 26a | Who will obtain informed consent or assent from potential trial participants or authorised surrogates, and how (see Item 32) | 11, 24 |
|  | 26b | Additional consent provisions for collection and use of participant data and biological specimens in ancillary studies, if applicable | N/A |
| Confidentiality | 27 | How personal information about potential and enrolled participants will be collected, shared, and maintained in order to protect confidentiality before, during, and after the trial | 13, 14, 20 |
| Declaration of interests | 28 | Financial and other competing interests for principal investigators for the overall trial and each study site | 24 |
| Access to data | 29 | Statement of who will have access to the final trial dataset, and disclosure of contractual agreements that limit such access for investigators | 24 |
| Ancillary and post-trial care | 30 | Provisions, if any, for ancillary and post-trial care, and for compensation to those who suffer harm from trial participation | 19 |
| Dissemination policy | 31a | Plans for investigators and sponsor to communicate trial results to participants, healthcare professionals, the public, and other relevant groups (eg, via publication, reporting in results databases, or other data sharing arrangements), including any publication restrictions | 19 |
|  | 31b | Authorship eligibility guidelines and any intended use of professional writers | 25 |
|  | 31c | Plans, if any, for granting public access to the full protocol, participant-level dataset, and statistical code. | NA |
| Appendices |  |  |  |
| Informed consent materials | 32 | Model consent form and other related documentation given to participants and authorised surrogates | 12 |
| Biological specimens | 33 | Plans for collection, laboratory evaluation, and storage of biological specimens for genetic or molecular analysis in the current trial and for future use in ancillary studies, if applicable | N/A |

*It is strongly recommended that this checklist be read in conjunction with the SPIRIT 2013 Explanation & Elaboration for important clarification on the items. Amendments to the protocol should be tracked and dated. The SPIRIT checklist is copyrighted by the SPIRIT Group under the Creative Commons “[Attribution-NonCommercial-NoDerivs 3.0 Unported](http://www.creativecommons.org/licenses/by-nc-nd/3.0/)” license.

**Supplementary Table 2.** Eligibility Criteria (exclusion, further details)

| Use of one or more prohibited treatments within specified timeframe, or not willing to avoid treatment for the duration of the study:   - - Oral contraceptive pill, or systemic HRT within the last 6 months^ⱡ^   - Anti-estrogen medication within the last 6 months   - Oral, intramuscular or intraarticular steroid within the last 3 months   - Intraarticular hyaluronan to a hand joint within the last 6 months   - Initiation of new oral analgesia within the last 4 weeks   - Initiation of glucosamine, chondroitin, hand exercises or other relevant non-pharmacological therapy within the last 6 weeks   - Hand surgery within the last 6 months, or planned within the next 6 months   - Medications likely to increase hepatic metabolism of study medication, including: St. John’s Wort; anti-convulsants (phenobarbital, phenytoin, carbamazepine, lamotrigine); some anti-infectives (i.e. rifampicin, rifabutin, nevirapine, efavirenz, ritonavir and nelfinavir)   Presence of one or more medical contraindications to the use of systemic hormonal replacement therapy:   - - In those aged 40-45 years, FSH <30 mIU/ml on screening blood test, i.e. non- confirmatory of menopausal status   - Any history of breast, endometrial, ovarian or skin cancer   - Any other history of other cancer within 5 years (except treated Basal Cell Carcinoma)   - Relevant breast issue on routine national breast screening in prior 3 years   - Undiagnosed genital bleeding, or untreated endometrial hyperplasia, active uterine fibroids or endometriosis   - Active or past history of venous thromboembolism (VTE) (including deep venous thrombosis, pulmonary embolism and retinal vein thrombosis), or at high risk of VTE (such as known thrombophilic disorders (such as Protein C, S or anti-thrombin deficiency) or presence of a strong family history of VTE^¥^)   - Active or past history of arterial thrombo-embolic disease (such as myocardial infarction, angina or stroke) or strong family history of stroke^¥^   - Clinically significant immobility   - Migraine or active epilepsy   - Uncontrolled hypertension (or diastolic pressure greater than 90 mmHg or systolic pressure greater than 145 mmHg at screening visit)   - Uncontrolled diabetes mellitus or uncontrolled hypertriglyceridaemia   - Body Mass Index BMI greater than 30   - Active malabsorption syndrome or clinically significant small bowel disease   - Acute liver disease, clinically significant abnormal liver function, active gallbladder disease or porphyria   - Clinically significant renal impairment   - Intolerance to lactose, fructose or glucose (including galactose intolerance, lactase deficiency, fructose intolerance, glucose-galactose malabsorption or sucrase-isomaltase insufficiency)   - Known sensitivity to either conjugated equine estrogens, bazedoxifene or the combination   ^ⱡ^Use of an intrauterine contraceptive device with progesterone local therapy (such as Mirena^TM^) or vaginal topical estrogen use (known low systemic absorption) are not exclusions to participation.  ^¥^Women with a first degree relative with a history of VTE, or other strong family history of VTE at the Investigators’ discretion. |
| --- |

**Supplementary Figure 1.** Flow Chart for Postmenopausal Bleeding
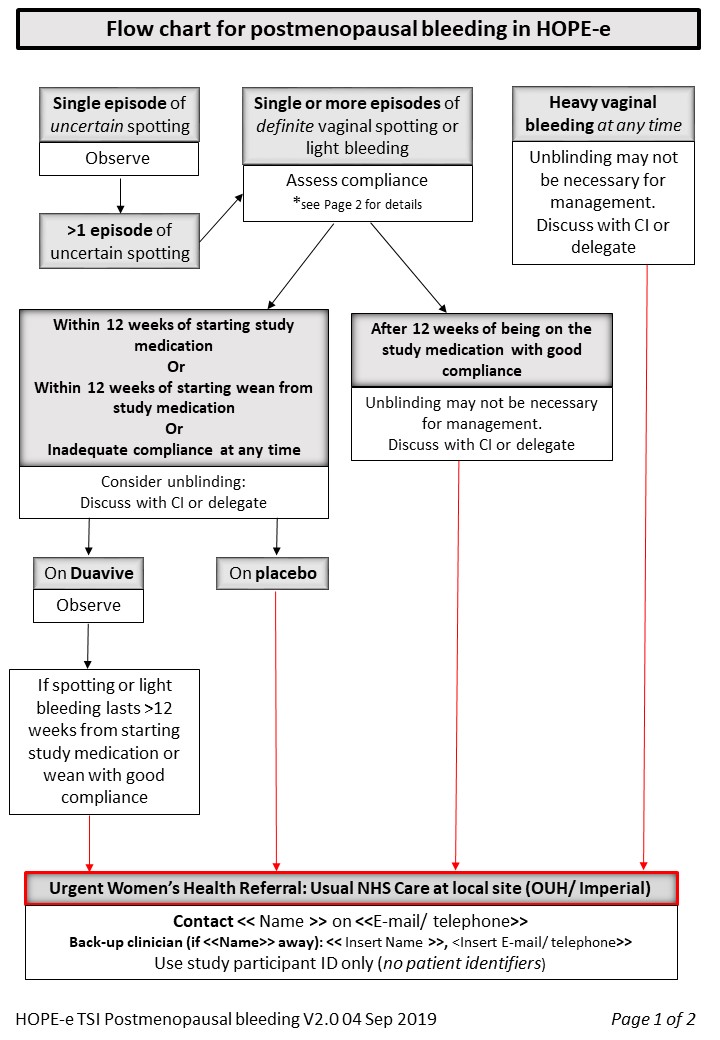


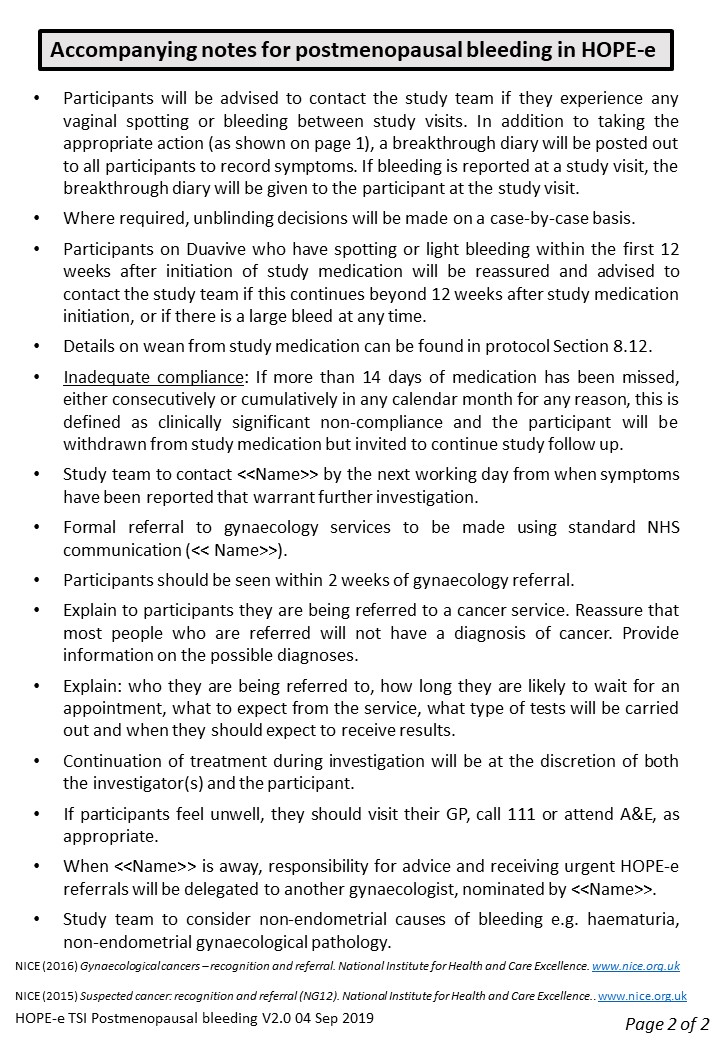


**Supplementary Table 3.** Protocol Amendment History*

| **Amendment No.** | **Protocol Version No.** | **Date issued** | **Details of Changes made including reason for change** |
| --- | --- | --- | --- |
| 4 | 4.0 | 30 Apr 2020 | Updates to Investigator list  Addition of Chelsea & Westminster Hospital as a secondary care Participant Identification Centre  Addition of Pulvertaft Hand Centre (University Hospitals of Derby and Burton NHS Foundation Trust) as a fourth research site  Removal of reference to specific sites in regards to ability to carry out out-of-hours unblinding via trials pharmacy |
| 3 | 3.0 | 06 Dec 2019 | Clarification to inclusion criteria (7.2) that the criteria related to use of an intrauterine contraceptive device with progesterone local therapy also includes women who were using this at the time of menopause.  Inclusion criteria updated to include at least 2, painful hand joints of any type (interphalangeal or base of thumbs). Patients with base of thumb OA only do not need to meet the American College of Rheumatology criteria.  Broadening of inclusion criteria to consider 'typical' average hand pain as well as average hand pain reported in the last 7 days at the screening visit.  Changes to recruitment strategies (8.1.1.):   - Broadening the method by which the GP database search will be carried out to include SMS messaging as well as posting study details to those identified by a database search. - Addition of Fortius Clinic as a secondary care Participant Identification Centre. - The addition of community advertising to the recruitment strategy (posters, flyers, social media).   A further stratification group (base of thumb OA only) has been added to Section 8.5 to align with the change to inclusion criteria.  Statistics section (11.1) updated to reflect the three stratification groups. |
| 2 | 2.0 | 20 Jun 2019 | The Inclusion criteria have been updated to include women who are using an intrauterine contraceptive device with progesterone local therapy (such as Mirena). These women, will need to meet the same inclusion criteria as those women who have undergone a hysterectomy because this group of women do not have regular periods and therefore 12 months of spontaneous amenorrhea is not a reliable method for determining menopause.  Blood volume at the Screening Visit increased from 20 ml to 25 ml. The hormone testing needs to be in a separate 5 ml SST II Gold top tube to the Autoantibody testing and 20 ml is not sufficient for this.  The optional study (to provide an additional sample of blood and urine) has been removed from the protocol because it comes under a separate Research Ethics Committee approval.  Section 10.2.1 has been updated detailing the process that should be followed if a participant experiences vaginal bleeding. This change is necessary because those participants who are on placebo and experience breakthrough bleeding should be referred for urgent investigation of post-menopausal bleeding as per NICE guidelines.  Section 8.5 (code breaking) has been modified to limit unnecessary unblinding of the Investigators. Also, in circumstances where unblinding occurs via local Trials Pharmacy, only an unblinding request form will be required to be sent to the pharmacist and not a log. The unblinding log will instead be updated by the central study team once the record of unblinding form has been received (this form does not reveal the treatment allocation unlike the unblinding request form).  Frimley Health NHS Foundation Trust has been added as a participant identification centre.  The list of centres where a participant at Faringdon may have their X-ray has been expanded to mention that the X-ray can be performed at a choice of several nearby centres.  Oxford Brookes hand clinic as a method of recruitment has been removed from because this clinic is no longer running.  References to Arthritis Research UK Centre for OA Pathogenesis have been changed to Centre for OA Pathogenesis Versus Arthritis in line with Arthritis Research UK’s new name ‘Versus Arthritis’.  Physical Examination has been added Visit 3 in the table in Appendix B of the protocol to be consistent with Section 8.7 of the protocol.  The study medication, Duavive, has not been ordered via the NHS Supply chain, Section 9.3 of the protocol has been updated to reflect this. |
| * Amendment 1 was a minor amendment which did not include amendment to protocol, therefore is not listed. | | | |
